# Life-stage dependent responses to microplastics and 2-methoxy-1,4-naphthoquinone in *Daphnia magna*

**DOI:** 10.1038/s41598-026-61016-5

**Published:** 2026-07-20

**Authors:** Julian Brehm, Jens G. P. Diller, Michael Schwarzer, Josef Breu, Christian Laforsch

**Affiliations:** 1 Animal Ecology I, Bayreuth Center for Ecology and Environmental Research (BayCEER), University of Bayreuth, 95447 Bayreuth, Germany; 2 Anorganic Chemistry I, University of Bayreuth, 95447 Bayreuth, Germany

**Keywords:** Microplastics, *Daphnia magna*, 2-MNQ, Invasive species, Freshwater ecosystems, Co-exposure, Ecology, Ecology, Environmental sciences

## Abstract

Multiple stressors threaten freshwater ecosystems, including microplastic pollution and invasive species. The invasive Himalayan balsam, *Impatiens glandulifera*, releases allelochemicals, such as 2-methoxy-1,4-naphthoquinone (2-MNQ), that can enter aquatic systems via runoff. While microplastics affect aquatic organisms and can alter the fate of chemical stressors, the effects of co-exposure with plant-derived allelochemicals remain largely unexplored. Using *Daphnia magna*, we investigated the single and combined effects of well-characterized microplastic fragments (PA 66 and PS; 500 and 1000 particles/mL) and 2-MNQ (0.75 mg/L) under chronic exposure, with kaolinite as a natural particle control. Ultraviolet–visible spectroscopy indicated that dissolved 2-MNQ concentrations declined over time in particle-free conditions, whereas the presence of particles influenced the detectable dissolved fraction. Biological responses differed across life stages. At primiparity, particle exposure reduced body length, whereas 2-MNQ and co-exposure showed no significant effects. In contrast, after 21 days, effects shifted towards 2-MNQ-containing treatments, which reduced body length and affected reproductive output, particularly during later broods. These findings demonstrate that the effects of microplastics and 2-MNQ are not static but shift over time, with particle-driven effects dominating early responses and combined effects becoming more relevant during prolonged exposure, potentially affecting long-term population dynamics.

**Supplementary Information:**

The online version contains supplementary material available at 10.1038/s41598-026-61016-5.

## Introduction

The environmental pollution caused by plastics and microplastic particles (MPs), proposed as plastic particles of 1 to < 1000 μm in size^1^, is considered one of the most widespread and long-lasting anthropogenic changes to the surface of our planet^2,3^. In recent years, an increasing number of studies have documented the presence of MPs in various freshwater systems worldwide. The detected particles vary in size, with particles in the lower micrometer range being particularly abundant, and differ considerably in shape and polymer type^4,5^.

Once MPs enter aquatic environments, filter-feeding organisms are particularly susceptible to MP exposure, as they can ingest a wide range of MP shapes and sizes and may bioaccumulate them^6^. In addition, when organisms are exposed to MPs, several adverse effects have been observed, such as oxidative stress, reduced reproduction, and morphological and molecular alterations^7–9^.

In addition to MP pollution, freshwater ecosystems are exposed to multiple stressors, including eutrophication, chemical contaminants, and non-native species^10,11^. Many of these pollutants originate from terrestrial environments and are transported into aquatic systems via runoff^12^, including plant-derived chemical substances released by terrestrial species^13^. Among these plant species is the Himalayan balsam, *Impatiens glandulifera*, an invasive species in Europe. It inhibits the growth of other plants by releasing allelopathic substances, which play a crucial role in its success in invasion^14^. This plant may also threaten freshwater ecosystems by forming large populations in riparian areas^15^ and releasing 2-methoxy-1,4-naphthoquinone (2-MNQ) through its roots or leaves, which subsequently enters freshwater ecosystems via rainfall runoff at concentrations as high as 12 mg/L^16,17^. Studies have shown that 2-MNQ has adverse effects on invertebrate communities^18,19^ and reduces somatic growth, offspring production, and defense capabilities in the filter-feeding crustacean *Daphnia magna*^20,21^.

As freshwater organisms are subjected to multiple interacting stressors that may act additively, synergistically, or antagonistically^11,22^, several studies have investigated the effects of MPs in multi-stressor scenarios. However, multiple-stressor studies with MPs have, up to now, mainly focused on temperature^23,24^, food availability^25,26^, metal compounds^27,28^, or chemical pollutants such as pesticides^29,30^. In this context, MPs can alter the toxicity of chemical stressors, e.g., by adsorption and desorption. However, this aspect has not yet been investigated in relation to chemical compounds released by invasive species. Hence, this study aims to investigate potential interactions between two major drivers of species decline and ecosystem degradation in freshwater ecosystems^22^: MP pollution and the impact of invasive species (2-MNQ from *I. glandulifera*) on the freshwater crustacean *D. magna*. In addition, by using two well-characterized MP polymer types and a natural particle control, we aim to rule out the effects of particle presence per se and elucidate whether potential effects depend on the MP polymer type.

## Material & methods

### *Daphnia magna* culture conditions

The laboratory-cultured *D. magna* clone BL2.2 was used in the experiments. This clone represents a well-established laboratory clone, originating from a small pond (Oud Meren) in a park in Leuven, Belgium, and has been maintained in the laboratory since 1997. Twice a year, reference toxicity tests with NaCl are performed in accordance with OECD Guideline 202^31^ and Santos et al. (2007)^32^ to validate culture sensitivity and health. The most recent test resulted in a 48 h EC_50_ value of 3.97 g/L NaCl. In the run-up to the chronic exposure experiment, 20 female animals were cultured in 1.5 L jars filled with 1 L vacuum-filtered (circular filters (retention range: 2–3 μm); ROTILABO Type 15 A, Carl Roth GmbH & Co. KG, Karlsruhe, Germany) M4 medium^33^. Animals were cultured in a climate chamber at 20 °C under a 16:8 h day (~ 800 lx)/night rhythm, including a 30-minute twilight period at dawn and dusk. *Daphnia* were fed *ad libitum* with the unicellular alga *Acutodesmus obliquus* three times a week, while the medium was renewed twice a week.

The first- and second-brood neonates were removed within 24 h after release, accompanied by medium renewal to avoid crowding effects. For experimental purposes, third brood parthenogenetic neonates (< 24 h) were used.

### 2-MNQ preparation

2-methoxy-1,4-naphthoquinone (2-MNQ; MW = 188.19 g/mol; log_Kow_ = 1.13) was purchased from Sigma-Aldrich (CAS number: 2348-82-5; Darmstadt, Germany). Dimethyl sulfoxide (DMSO) (Bernd Kraft GmbH, Duisburg, Germany) was used as a solvent. The concentrations used in the experiment were 0.75 mg/L 2-MNQ and 0.1 mL/L DMSO, and the solution was freshly prepared before each medium exchange. The concentration was selected based on Diller et al. (2022), as adverse effects in the organisms were already observed at this concentration^20^.

### Particle characteristics/preparation

Irregular MP fragments with a median size of < 20 μm of polystyrene (PS) (158 N/L – INEOS Styrolution Group GmbH, Frankfurt am Main, Germany, density: 1.04 g/cm^3^, d_90_: 35.08 μm) and polyamide (PA 66) (Ultramid A27 E – BASF SE, Ludwigshafen am Rhein, Germany, density: 1.14 g/cm^3^, d_90_: 44.50 μm) were used. Fragments were obtained using an ultra-centrifugal mill (ZM200, Retsch GmbH, Haan, Germany) at 18,000 rpm (corresponding to approximately 18,112 × g based on a rotor radius of 5 cm), followed by air-jet sieving. In addition, laser diffraction (LD) and dynamic image analyses (DIA) were performed using FLOWSYNC (Microtrac MRB, Montgomeryville and York, USA) to obtain information on particle size and shape (see Figure S1 A & B for more details). Particles of the same polymer types have been characterized in-depth^34^. For example, residual monomer (styrene) was found in PS, and piperazine was found in PA 66. As natural control particles, kaolinite was used (d_96_: < 10 μm; see Figure S1 C; density: ~ 2.6 g/cm³, Gebrüder Dorfner GmbH & Co., Hirschau, Germany).

To obtain further information on size and morphology of the particles, scanning electron micrographs of the MP and kaolinite particles were obtained using a JEOL JSM-IT500 Scanning Electron Microscope (SEM, JEOL Ltd., Japan). Particles were fixed to aluminum stubs (∅ 12 mm, Plano GmbH, Wetzlar, Germany) on conductive carbon pads (G3347, Plano GmbH, Wetzlar, Germany) and dried in a desiccator for 24 h. Before analysis, the samples were coated with 2 nm of carbon and 2 nm of platinum (Leica EM ACE600, Leica Microsystems GmbH, Germany).

To facilitate homogenization, MP particles and kaolinite were suspended in M4 medium (0.5 mg/mL) and shaken for 48 h^35^. Stock suspensions were then stored at 4 °C and stirred before dilution. Particle concentrations of stock suspensions were evaluated using a hemocytometer (Neubauer Improved, Brand GmbH & Co. KG., Wertheim, Germany).

### Exposure to *D. magna*

The experiment was conducted in a climate chamber at 20 ± 0.5 °C and a 16:8 h day/night rhythm, with a 30-minute twilight period at dawn and dusk. The testing procedure was based on the OECD guideline “*Daphnia magna* Reproduction Test 211”^36^. Third brood neonates (< 24 h) (*n* = 15/treatment) were individually placed in 100 mL glass beakers containing 50 mL of vacuum-filtrated M4 medium with varying concentrations of the complementary treatment (Table 1).

**Table 1:** Overview of the treatments used in the experiment.

Concentrations	Treatment ID
-	Control
500 Particles/mL kaolinite	Kaolinite low
1000 Particles/mL kaolinite	Kaolinite high
500 Particles/mL PA 66	PA low
1000 Particles/mL PA 66	PA high
500 Particles/mL PS	PS low
1000 Particles/mL PS	PS high
0.1 ml/L DMSO	DMSO
0.1 ml/L DMSO, 0.75 mg/L 2-MNQ	2-MNQ
1000 Particles/mL kaolinite, 0.1 ml/L DMSO, 0.75 mg/L 2-MNQ	Kaolinite high + 2-MNQ
500 Particles/mL PA 66, 0.1 ml/L DMSO, 0.75 mg/L 2-MNQ	PA low + 2-MNQ
1000 Particles/mL PA 66, 0.1 ml/L DMSO, 0.75 mg/L 2-MNQ	PA high + 2-MNQ
500 Particles/mL PS, 0.1 ml/L DMSO, 0.75 mg/L 2-MNQ	PS low + 2-MNQ
1000 Particles/mL PS, 0.1 ml/L DMSO, 0.75 mg/L 2-MNQ	PS high + 2-MNQ

**Fig. 1:**
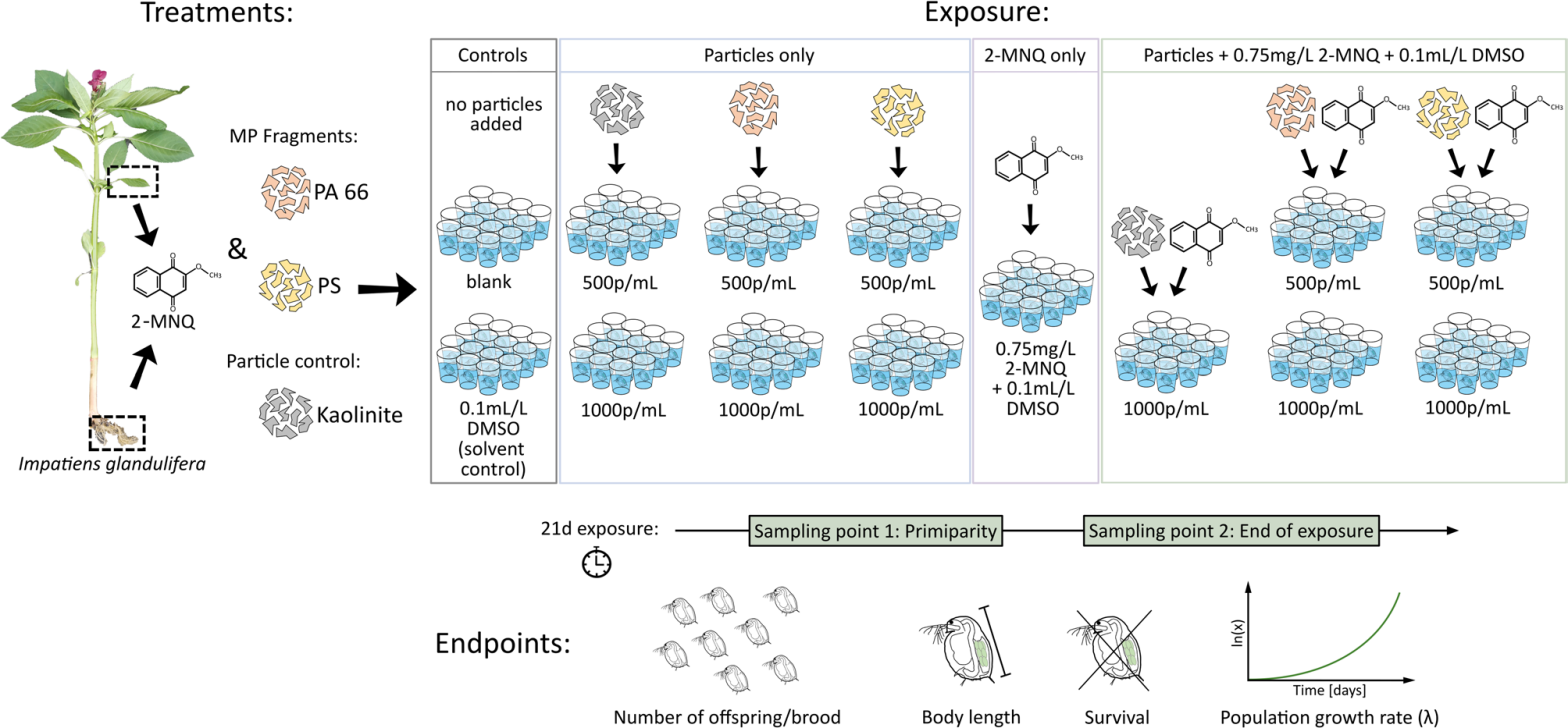
Experimental design of the chronic exposure study. Overview of the experimental setup investigating single and combined effects of microplastic (MP) fragments (polyamide 66 (PA 66) and polystyrene (PS)), natural control particles (kaolinite), and the plant-derived allelochemical 2-methoxy-1,4-naphthoquinone (2-MNQ) on *D. magna*. Neonates (< 24 h old) were exposed for 21 days. Endpoints were assessed at primiparity and at the end of exposure, including body length, survival, reproduction, brood timing, and population growth rate (λ).

According to the guideline, the medium was renewed every second day, accompanied by pH and O_2_-level measurements of both the fresh and old medium. Throughout the experiment, dissolved oxygen concentrations remained above 7 mg/L, and pH values were stable at 7.9 ± 0.5 across all treatments. Animals were fed daily with 2 mg C/L of the unicellular algae *A. obliquus*. Life-history traits, including survival and reproduction, were monitored daily throughout the experiment. Upon reaching primiparity (mean time to primiparity: 6.3–8.9 days, depending on treatment; Table S1) and at the end of the experiment (at day 21), the body length was measured under a stereomicroscope (Leica M50 with cold light source Leica KL 300 LED, Leica Microsystems, Germany), equipped with a digital camera for microscopy (Olympus DP26 camera, Olympus Deutschland GmbH, Hamburg, Germany) and a digital image analysis system (CellSens Dimension v. 1.11, Olympus Deutschland GmbH, Hamburg, Germany). Body length was measured as the distance from the upper edge of the compound eye to the base of the apical spine. An overview of the experimental design, including all treatments, sampling points, and measured endpoints, is provided in Fig. 1.

### Determination of dissolved 2-MNQ concentrations in experimental beakers

To assess changes in dissolved 2-MNQ concentrations during the exposure period, UV–vis spectroscopy was performed. First, the absorption spectrum of 2-MNQ was characterized by measuring M4 medium containing 100 µl/L DMSO and 0.75 mg/L 2-MNQ in UV-transparent 96-well plates (UV-Star Microplates, Greiner Bio-One GmbH, Frickenhausen, Germany), using a Plate Reader (VARIOSKAN LUX, Thermo Scientific, Waltham, USA). Absorbance spectra were recorded from 200 to 400 nm in 1 nm intervals, and the absorption maximum at 220 nm was selected for quantification. A calibration curve was established using defined 2-MNQ concentrations ranging from 0.015 mg/L to 1.5 mg/L (see Figure SI 2).

To determine changes in dissolved 2-MNQ concentrations under exposure conditions, separate experimental beakers were prepared following the same setup as the chronic experiment, including the respective particle treatments, but without adding *Daphnia* or algae to avoid biological uptake and degradation (*n* = 4 per treatment). Samples (2 mL) were collected after 0, 24, and 48 h, corresponding to the medium renewal interval during the chronic exposure experiment. Samples were filtered through 0.45 μm syringe filters (Rotilabo, Carl Roth GmbH + Co. KG, Karlsruhe, Germany) to remove particles and determine the remaining dissolved 2-MNQ fraction. Absorbance was measured in duplicate at 220 nm, with treatments blank corrected against its respective (filtered) particle-containing control without 2-MNQ. Dissolved 2-MNQ concentrations were calculated based on the calibration curve (y = 0,2494x + 0,0021; R^2^: 0.99).

### Statistical methods

To test the impact of MPs and 2-MNQ on *D. magna* life history and morphological parameters, statistical models were selected based on the respective data structures and distributions. Model assumptions were assessed using model-specific diagnostic approaches. For generalized linear models (GLMs), assumptions were evaluated using simulated residual diagnostics with the functions `simulateResiduals` and `testDispersion` from the `DHARMa` package^37^.

For generalized least squares (GLS) models, residual distributions were assessed visually using residual and Q–Q plots, and residual normality was additionally evaluated using Shapiro–Wilk tests. Variance heterogeneity was addressed by comparing GLS models assuming homogeneous residual variance with models allowing treatment-specific variances using the `varIdent` variance structure (`nlme` package^38^. Unless stated otherwise, pairwise comparisons were performed using estimated marginal means (`emmeans` package^39^ with Holm correction for multiple testing. Statistical significance was accepted at α = 0.05. All analyses were conducted in R version 4.4.2^40^.

Total offspring production per individual over the experimental period was analyzed using GLS models. Treatment was included as fixed effect. Overall treatment effects were assessed using model-based analysis of variance. Treatment differences and comparisons with the control were evaluated as described above.

The number of neonates per brood was analyzed using GLMs with a Poisson distribution and log-link function. Since reproductive output was measured repeatedly across broods for individual animals, generalized linear mixed models including IDs as random intercept were initially evaluated. However, the estimated random-effect variance was negligible, and the simpler GLM was therefore retained as the final model. Treatment, brood number, and their interaction were included as fixed effects. Overall effects were assessed using `Anova` (`car` package). Treatment differences within individual broods were evaluated as described above.

To evaluate whether the effects of 2-MNQ depended on the presence and type of particles, a factorial Poisson GLM was fitted, including particle type, 2-MNQ exposure, brood number, and their interactions as explanatory variables. Particle type was defined as no particle, kaolinite, PA 66, or PS, and 2-MNQ exposure was included as a binary factor (presence/absence). This analysis was used to test for statistical particle × 2-MNQ interactions but was not intended as a formal mixture toxicity assessment according to concentration addition or independent action models.

Body length at primiparity and at the end of the experiment (day 21) was analyzed using GLS models. Models assuming homogeneous residual variance were compared with models allowing treatment-specific variances (`varIdent`), and the best-fitting model was selected based on Akaike’s Information Criterion (AIC). Treatment effects were assessed separately for both developmental stages using model-based analysis of variance. Pairwise treatment differences were evaluated as described above.

To investigate whether the effect of 2-MNQ exposure on body length depended on developmental stage and particle exposure, an additional factorial GLS model was fitted including particle type, 2-MNQ exposure, time point, and their interactions.

## Results

### SEM micrographs of kaolinite and MP particles

**Fig. 2:**
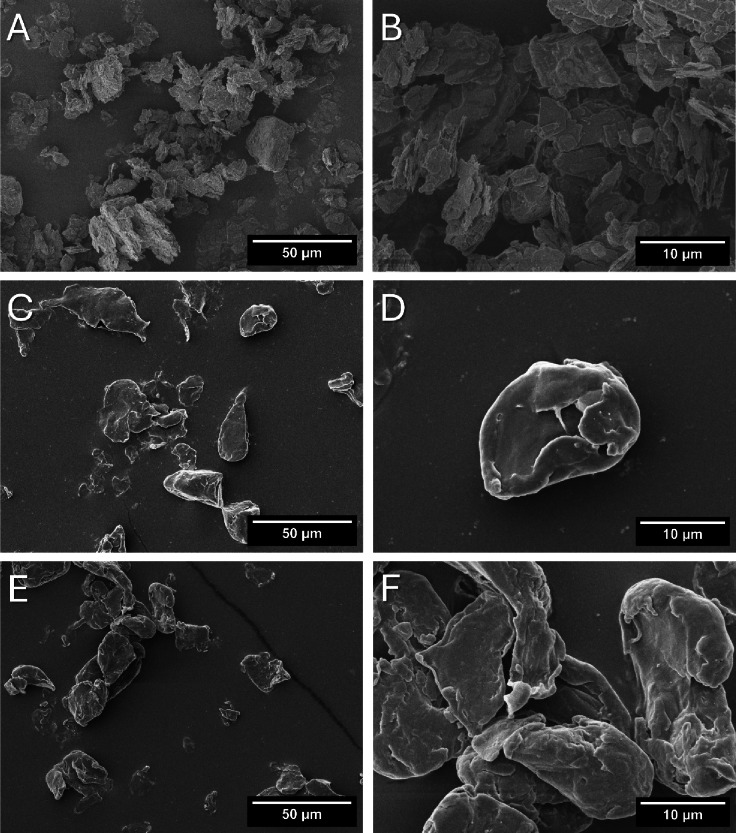
Scanning electron microscopy (SEM) micrographs of kaolinite and microplastic particles (MPs). (**A**) kaolinite, 700x magnification; (**B**) kaolinite, 3000x magnification. (**C**) Polyamide 66 (PA 66), 700x magnification; (**D**) PA 66, 3000x magnification. (**E**) Polystyrene (PS), 700x magnification; (**F**) PS, 3000x magnification.

SEM analysis was used to characterize the morphology of kaolinite and MP particles (Fig. 2). Kaolinite particles (d_96_ < 10 μm) were generally smaller than the MP particles and exhibited a plate-like morphology with sharp edges, resembling slate slabs (Fig. 2A, B). In contrast, PS (d_90_: 35.08 μm) and PA 66 particles (d_90_: 44.50 μm) appeared more irregular to rounded in shape and exhibited smoother edges compared with kaolinite particles (Fig. 2C–F). MPs of both polymers showed comparable overall morphologies and size ranges, consistent with LD/DIA measurements presented in Figure SI 1.

### 2-MNQ binding and stability

**Fig. 3:**
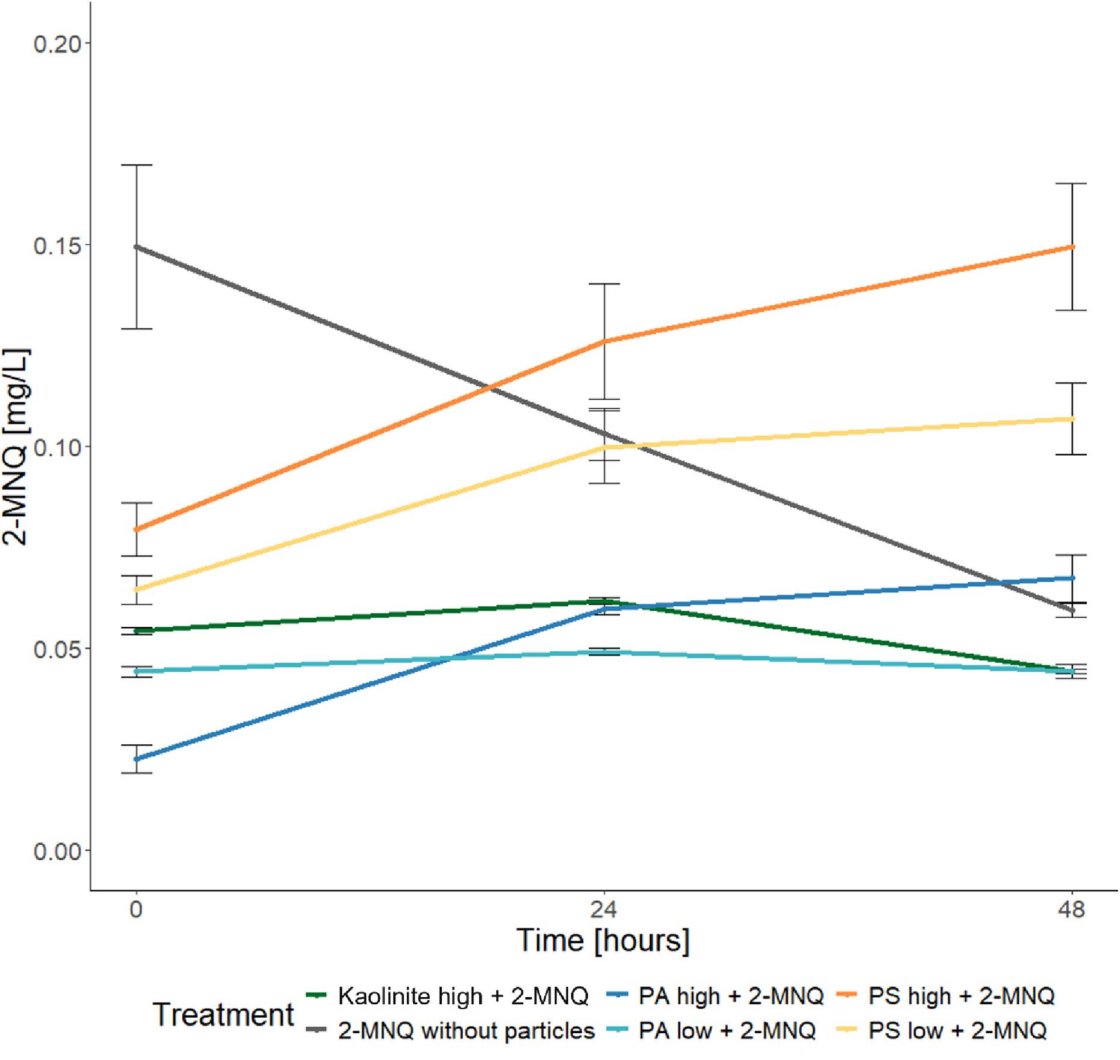
2-methoxy-1,4-naphthoquinone (2-MNQ) concentrations in mg/L measured at 0, 24, and 48 h after incubation via Ultraviolet–visible spectroscopy (UV-Vis) at 220 nm. Error bars indicate mean ± standard error (SE) (*n* = 4).

The concentration of dissolved 2-MNQ in pure M4 medium decreased almost linearly over 48 h (Fig. 3). In particle-containing treatments, dissolved 2-MNQ concentrations at t = 0 were substantially lower than in particle-free medium. While concentrations remained relatively constant in the kaolinite treatment, they increased over time in the MP treatments, with patterns depending on polymer type and particle concentration. The reduced concentrations at t = 0 indicate that the presence of particles affected the detectable dissolved fraction after filtration. However, because particle-associated 2-MNQ was not quantified directly, the present data do not allow us to distinguish between adsorption to particles, filtration-related losses, or other matrix effects (additional information, SI Figures S3 and S4). The highest dissolved 2-MNQ concentration after 48 h was observed in the treatment with high PS MP concentrations. The discrepancy between nominal (0.75 mg/L) and measured concentrations may be related to the filtration step required to remove particles prior to analysis.

### Chronic exposure

Control mortality remained below the 20% threshold defined in OECD Test Guideline 211. Surviving control animals produced 50.25 ± 2.63 live offspring per individual over the 21-day exposure period. Although reproductive output was below the OECD recommendation of 60 offspring per surviving control female, all treatments were conducted under identical experimental conditions, allowing robust relative comparisons among treatments.

### Life history - Reproduction

**Fig. 4:**
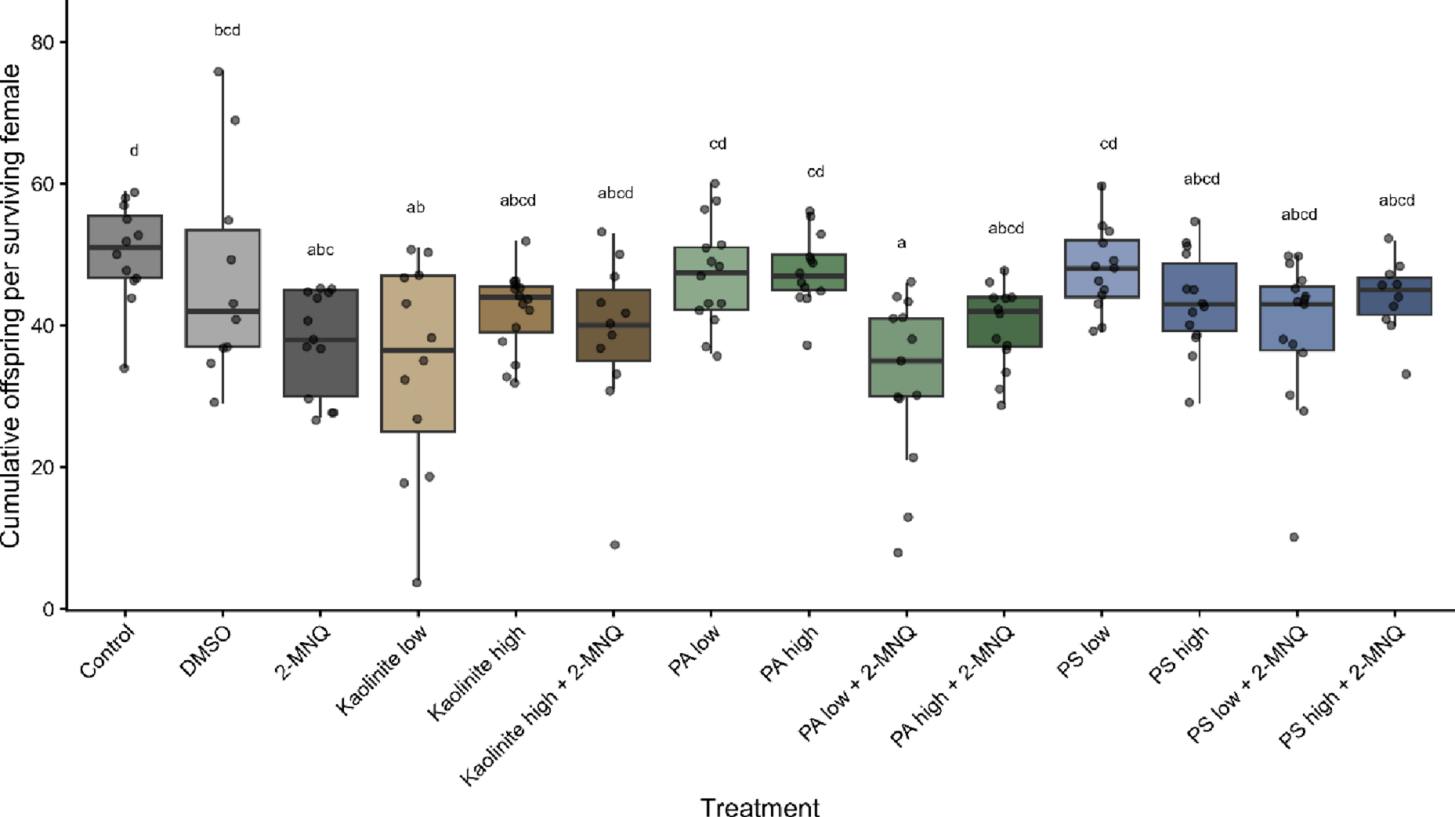
Total offspring production of *D. magna*, expressed as the cumulative number of offspring per individual over the experimental period under chronic exposure to microplastic particles (MPs) (polystyrene (PS), polyamide 66 (PA 66)), kaolinite, and 2-methoxy-1,4-naphthoquinone (2-MNQ), applied individually and in combination. Boxes represent the interquartile range, horizontal lines indicate medians, and grey points show individual replicates. Treatment effects were analyzed using generalized least squares (GLS) models, followed by pairwise comparisons based on estimated marginal means with Holm correction for multiple testing. Different letters indicate significant differences among treatments (*p* < 0.05).

Reproduction was analyzed as cumulative offspring production per individual and at the level of individual broods to account for temporal variation in reproductive output. Total offspring production differed significantly among treatments (GLS, F₁₃,₁₆₄ = 4.50, *p* < 0.0001; Fig. 4). Compared with the control (50.25 ± 2.63 offspring per individual), the strongest reduction was observed after combined exposure to PA low + 2-MNQ (32.31 ± 2.53 offspring per individual), corresponding to a 35.7% decrease. Reduced cumulative offspring production was also observed in Kaolinite low (34.25 ± 2.63; −31.8%), 2-MNQ alone (37.69 ± 2.53; −25.0%), and Kaolinite high + 2-MNQ treatments (38.55 ± 2.75; −23.3%). In contrast, MP-only treatments showed only minor effects on total offspring production, with PA 66 and PS treatments ranging between 43.43 and 47.77 offspring per individual.

To further investigate whether the effect of 2-MNQ depended on particle exposure, a factorial GLS model was applied. This analysis revealed significant effects of 2-MNQ exposure (F_1,133_ = 12.55, *p* < 0.001), particle type (F_2,133_ = 5.33, *p* = 0.006), and particle concentration (F_1,133_ = 5.08, *p* = 0.026). Importantly, the effect of 2-MNQ depended on particle type, as indicated by a significant 2-MNQ × particle type interaction (F_2,133_ = 4.08, *p* = 0.019). This interaction was mainly driven by PA 66, where the addition of 2-MNQ reduced offspring production by 11.52 offspring per individual compared with PA 66 exposure alone (*p* < 0.001). In contrast, the effects of adding 2-MNQ were weaker and not statistically significant for PS (− 3.88 offspring, *p* = 0.119) and kaolinite (− 7.37 offspring, *p* = 0.061).

**Fig. 5:**
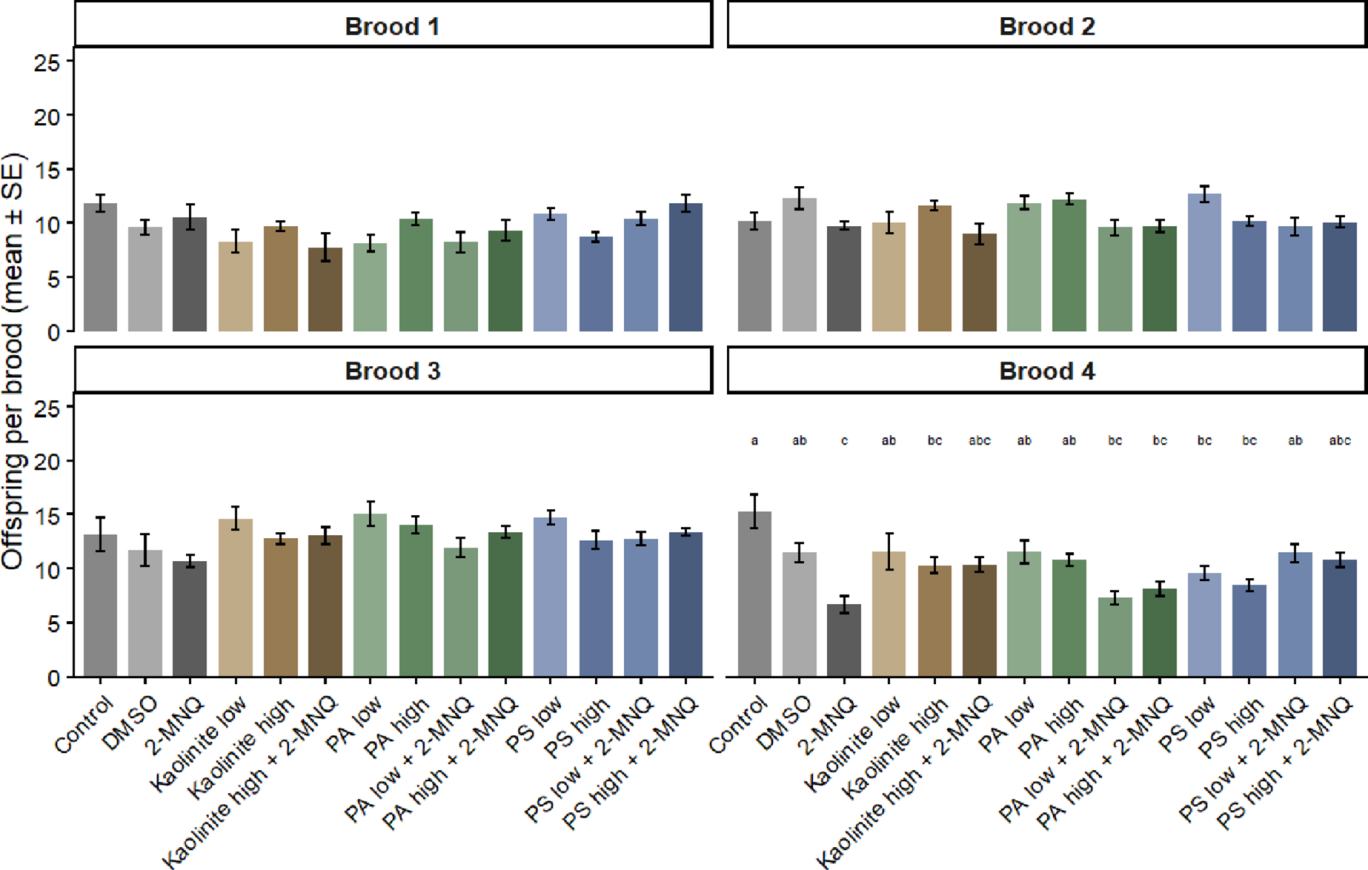
Offspring numbers of *D. magna* per brood (Broods 1–4), expressed as mean ± standard error (SE), under exposure to microplastics (MPs) (polystyrene (PS), polyamide 66 (PA 66)), kaolinite, and 2-methoxy-1,4-naphthoquinone (2-MNQ), applied individually and in combination. Treatments are grouped by particle type and concentration (low, high). Bars represent brood-specific mean offspring production per individual. Treatment effects were analyzed using a Poisson generalized linear model followed by estimated marginal means with Holm correction for multiple comparisons. Different letters indicate significant differences among treatments within each brood (*p* < 0.05).

When analyzing offspring production across individual broods, reproductive responses differed significantly among treatments and changed over time (Fig. 5). A Poisson GLM revealed significant effects of treatment (χ² = 62.17, df = 13, *p* < 0.001), brood (χ² = 100.63, df = 3, *p* < 0.001), and a treatment × brood interaction (χ² = 78.84, df = 39, *p* < 0.001), indicating that treatment effects depended on reproductive stage. Differences among treatments were weak during the first three broods, whereas pronounced effects emerged during the fourth brood. In Brood 4, control animals produced on average 15.3 ± 1.7 offspring, whereas exposure to 2-MNQ alone reduced offspring production by approximately 56% (6.7 ± 0.9 offspring). Similarly strong reductions were observed for PA low + 2-MNQ (7.4 ± 0.8 offspring; −52%), PA high + 2-MNQ (8.2 ± 1.0 offspring; −46%), and PS high (8.5 ± 0.8 offspring; −45%). Estimated marginal means with Holm-adjusted multiple comparisons indicated that treatment differences were primarily attributable to reduced reproductive output in later broods.

A factorial Poisson GLM further revealed a significant particle × 2-MNQ × brood interaction (χ² = 31.39, df = 9, *p* < 0.001), indicating that interaction effects varied across reproductive stages.

### Morphological parameters - Body length

Time to primiparity, defined as the first appearance of eggs in the brood chamber, varied slightly among treatments and ranged from 6.29 ± 0.16 days (PS high) to 7.92 ± 0.31 days (Kaolinite high + 2-MNQ) (Table S1). Accordingly, body length was measured individually for each organism at primiparity and again at the end of the experiment (day 21).

**Fig. 6:**
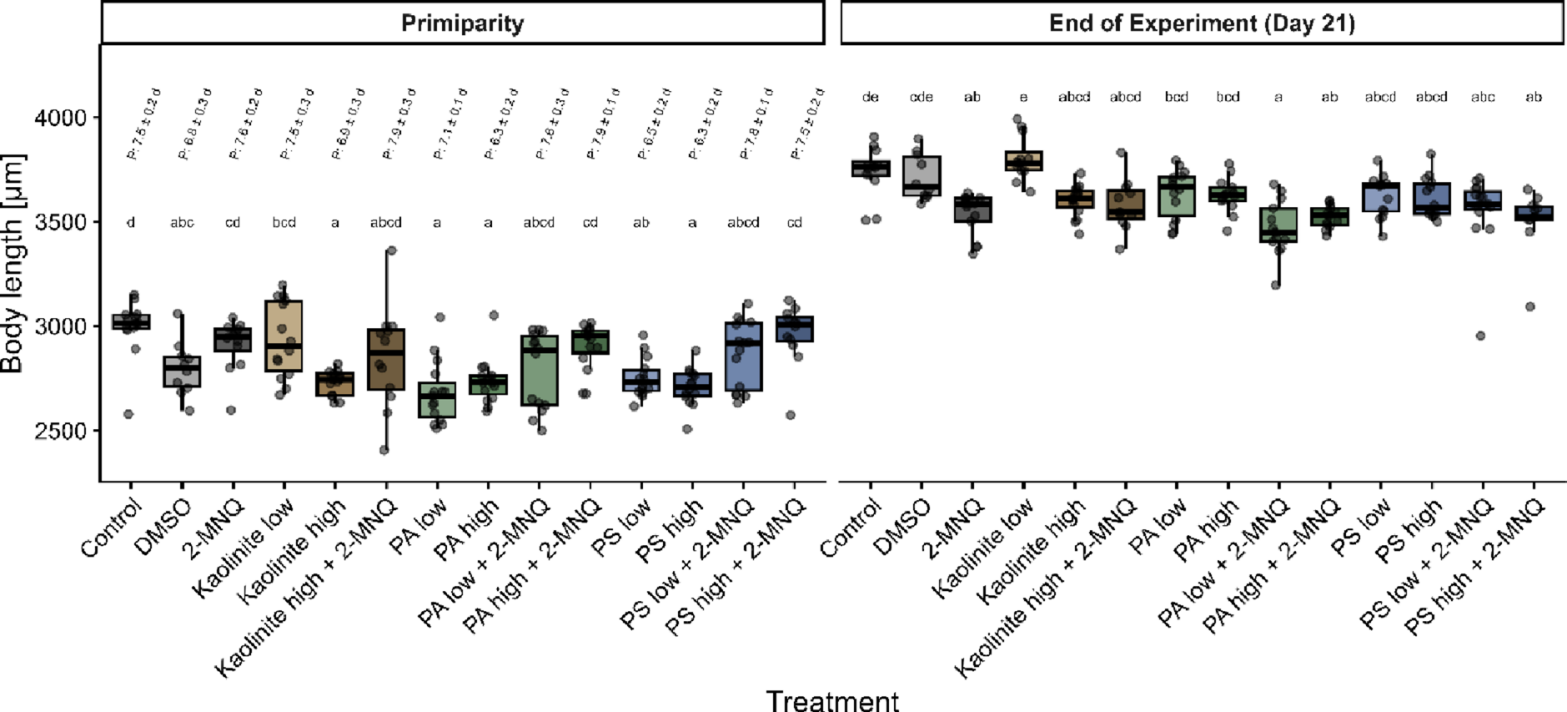
Body length of *D. magna* at primiparity and at the end of the experiment (Day 21) following chronic exposure to microplastics (MPs; polystyrene (PS), polyamide 66 (PA 66)), kaolinite, and 2-methoxy-1,4-naphthoquinone (2-MNQ), applied individually and in combination. Boxplots show the median, interquartile range (IQR), and 1.5 × IQR, while individual data points are displayed as jittered dots. Body length at primiparity was measured individually at the developmental stage when animals first carried eggs in the brood chamber. Values in the primiparity panel indicate the mean day of primiparity ± standard error (SE) for each treatment group. Body length was analyzed using generalized least squares (GLS) models. Different letters indicate significant differences among treatments within each time point based on estimated marginal means with Holm correction for multiple comparisons (*p* < 0.05).

Body length differed significantly among treatments both at primiparity (GLS: F₁₃,₁₇₄ = 9.54, *p* < 0.0001) and at the end of the experiment (GLS: F₁₃,₁₆₅ = 9.29, *p* < 0.0001) (Fig. 6). Treatment effects differed markedly between time points. At primiparity, body length was mainly reduced in particle-exposed animals, whereas exposure to 2-MNQ alone did not significantly affect body size. Compared to the control (2995 ± 39 μm), significant reductions were observed after exposure to PA 66 fragments (low: 2681 ± 39 μm; −10.5%; high: 2737 ± 28 μm, − 8.6%), PS fragments (low: 2752 ± 26 μm, − 8.1%; high: 2709 ± 24 μm, − 9.5%), and high kaolinite concentrations (2729 ± 17 μm, − 8.9%). In contrast, Kaolinite low and 2-MNQ alone did not significantly affect body length at this developmental stage. Although PA low + 2-MNQ remained significantly reduced compared with the control (2790 ± 49 μm; -6.8%), combined exposure treatments generally showed weaker reductions than particle-only treatments at primiparity.

At the end of the experiment, the observed response patterns shifted from primarily particle-driven effects towards stronger effects of 2-MNQ-containing treatments. Relative to the control (3740 ± 35 μm), exposure to 2-MNQ alone significantly reduced body length (3536 ± 28 μm; -5.4%). Similarly, combined exposure to MPs and 2-MNQ consistently resulted in reduced body length, with significant reductions observed for PA low + 2-MNQ (3469 ± 32 μm, − 7.2%), PA high + 2-MNQ (3525 ± 32 μm, − 5.8%), PS low + 2-MNQ (3556 ± 30 μm, − 4.9%), and PS high + 2-MNQ (3507 ± 35 μm, − 6.2%). In contrast, particle-only effects were less pronounced at day 21, with only PS high (3613 ± 31 μm, − 3.4%) and Kaolinite high (3603 ± 30 μm, − 3.7%) remaining significantly different from the control. The shift in response patterns was supported by a significant 2-MNQ × time point interaction (GLS: χ² = 72.67, *p* < 0.0001), indicating that the effect of 2-MNQ exposure on body length depended on developmental stage.

## Discussion

We did not observe increased mortality in *D. magna* during chronic exposure to control particles, MP, 2-MNQ, or their combination. However, sublethal adverse effects on life history and morphology were detected.

### Particle-dependent effects

Differences in body length among kaolinite-, MP-, and control-exposed animals indicate that particle effects were not solely related to particle presence but depended on particle characteristics such as polymer type, concentration, or surface properties, and may additionally be influenced by residual monomers^41^ and other chemicals or additives present in the polymers^42^. The MP fragments we used have been shown to contain residues of other chemicals, such as piperazine in PA 66 and the monomer styrene in PS^34^. Both chemicals are known to be toxic to daphnids, with piperazine having an EC_50_ of 105.4 mg/L in *D. magna*^43^ and styrene having an EC_50_ of 9 mg/L in *D. magna*^41^. Therefore, incorporated chemicals and residual monomers in polymers may be the missing link that explains the effects of MPs beyond interference with feeding^34,44^.

At primiparity, particle-only exposures resulted in reduced body length, particularly for kaolinite at high concentrations as well as for PA 66 and PS. After 21 days, particle-only effects on body length became less pronounced, indicating that the relative importance of particle exposure and chemical co-exposure changed over time. This suggests that daphnids may partially compensate for early-life stress at older ages, potentially due to increased stress tolerance in later life stages^45,46^, as also reported for PS MP exposure^47^. Thus, under the tested conditions, MP exposure did not result in persistent reductions in body length, indicating a capacity of the animals to recover over time.

Although particle-related effects on body length became less pronounced over time, reproductive responses remained affected in several treatments. This apparent divergence between body size and reproduction may reflect shifts in energy allocation under chronic stress. According to energy allocation frameworks, organisms have a limited energy budget that must be distributed among competing biological processes, including growth, reproduction, maintenance, and stress responses^48^. In *Daphnia*, trade-offs between somatic growth and reproduction are well documented and are thought to play an important role in responses to environmental stress. For example, altered resource allocation between growth and reproduction has been reported under food limitation and contaminant exposure, including heavy metals and organic pollutants^49,50^. Such stress-induced resource allocations between growth and reproduction are often associated with altered physiological energetics and increased energetic demands for maintenance processes, including detoxification, cellular repair, and stress defense mechanisms, potentially reducing the energy available for growth or reproduction^48,50^. Similar energy allocation shifts have recently been reported under combined MP and heavy metal exposure, where effects on feeding, somatic growth, and reproductive investment differed across endpoints^51^. Therefore, the reduced reproductive output observed in the present study, particularly during later reproductive stages, may reflect changes in energy allocation associated with chronic exposure rather than being solely explained by direct effects on reproduction.

Reproductive responses further emphasize the time-dependent nature of the observed effects. While total offspring production differed among treatments, these effects were not uniform across broods, as indicated by a significant treatment × brood interaction. In particular, treatment effects became more pronounced in the fourth brood, indicating that the influence of MPs depends on the reproductive stage and duration of exposure. Our findings suggest a concentration- and polymer-type-dependent pattern in the observed effects, highlighting that MPs should not be viewed as a uniform contaminant but rather as a multidimensional set of particles with diverse physicochemical properties, exerting effects depending, for example, on polymer type^52^. These differences may contribute to particle-specific biological responses.

### 2-MNQ-dependent effects

At primiparity, 2-MNQ-exposed animals exhibited no differences in body length compared to the control and solvent-control-treated animals. However, adverse effects became apparent at the end of the experiment, where both reduced body length and reduced reproductive output were observed. This time-dependent response suggests that organismal effects of 2-MNQ may depend on exposure duration, although internal 2-MNQ concentrations and potential accumulation in *D. magna* tissues were not measured in the present study. Naphthoquinones, such as 2-MNQ, can act through two major mechanisms: as prooxidants, reducing oxygen to reactive oxygen species, and as electrophiles, forming covalent bonds with tissue nucleophiles. This may affect various cell signaling pathways that promote and protect against inflammatory responses and cell damage^53,54^, potentially leading to the observed adverse effects.

### Co-exposure effects of particles and 2-MNQ

Under co-exposure conditions, dissolved 2-MNQ concentrations changed when particles were present. However, because particle-associated 2-MNQ was not directly quantified, it is not possible to distinguish between adsorption onto particles, changes in chemical stability, methodological effects related to filtration, or differences in bioavailability. Therefore, interactions between particles and 2-MNQ should be considered as potential explanations rather than confirmed mechanisms. In addition, the algae present during the chronic exposure represent an additional component of our exposure system. Algal cells can interact with both suspended particles and dissolved or particle-associated contaminants, thereby potentially modifying the distribution of 2-MNQ and MP and altering their bioavailability in the exposure medium. For filter-feeding organisms such as *Daphnia*, such interactions may influence exposure not only through changes in freely dissolved contaminant concentrations but also through ingestion-mediated pathways when contaminants or particles are associated with algal food. Previous studies have shown that algae can influence MP uptake^55^ and contaminant bioavailability^56^ in aquatic organisms. However, the direction and magnitude of these effects strongly depend on exposure conditions, including contaminant properties, particle characteristics, exposure duration, and food availability^57^. Thus, algae should be considered as an additional factor influencing MP and contaminant exposure dynamics in chronic tests, although their specific contribution was not quantified in the present study.

The biological effects of co-exposure depended strongly on exposure duration and endpoint. At primiparity, body length reductions were mainly associated with particle exposure, whereas effects of 2-MNQ-containing treatments became more apparent after prolonged exposure, particularly affecting body length after 21 days and reproductive output in the 4th brood. This temporal shift indicates that single- and combined-stressor effects are not static but may depend on developmental stage and exposure duration. Significant particle × 2-MNQ interactions were observed for reproductive endpoints, indicating that the effect of 2-MNQ depended on the co-occurring particle type. The strongest interaction was observed for PA 66, where combined exposure with 2-MNQ resulted in reduced reproductive output compared with PA 66 exposure alone.

One possible explanation is that particles may have influenced the exposure dynamics of 2-MNQ over time, for example, by altering its partitioning between dissolved and particle-associated phases or by modifying ingestion-related exposure pathways, as described for other stressors^58–60^. Interestingly, despite similar indications of reduced dissolved 2-MNQ concentrations in kaolinite treatments, no comparable enhancement of 2-MNQ effects was observed in kaolinite co-exposure, suggesting that interactions between 2-MNQ and MPs may differ from those with natural particles (i.e., kaolinite). However, direct measurements of particle-associated 2-MNQ concentrations and internal concentrations in *Daphnia* are required to evaluate these mechanisms.

### Environmental Relevance and Implications

The pollutant concentrations used in this study may fall within the range reported in environmental studies, although direct comparability remains limited. Mean 2-MNQ concentrations in rainfall water sampled from *I. glandulifera* leaves reached 12.21 ± 3.01 mg/L ^16^, indicating the potential for runoff into water bodies. However, the environmental relevance of the applied MP concentrations is more difficult to assess, especially in the small size classes we used. Direct comparisons between MP concentrations used in laboratory studies and those found in the environment remain challenging, particularly for small MPs, because reported concentrations depend strongly on sampling strategies, analytical methods, and the size range considered^61^. Nevertheless, measurements of MPs in freshwater demonstrate that small MPs are ubiquitous in aquatic ecosystems^62,63^. Consequently, the exposure of planktonic organisms such as *Daphnia* to small MP fragments is likely, but the magnitude of this exposure remains difficult to assess.

Although no increase in mortality was observed, chronic exposure to MPs, 2-MNQ, and their combinations affected key life-history traits, including body size and reproductive output. Such sublethal responses are particularly relevant to zooplankton, as changes in growth and reproduction can influence population dynamics over longer timescales and have knock-on effects within freshwater ecosystems^64,65^. In line with this, calculated population growth rates showed treatment-dependent variation, although all treatments maintained positive population growth under the laboratory conditions tested. These findings indicate that survival alone may underestimate the ecological consequences of chronic exposure and underscore the importance of integrating multiple life-history endpoints and time points^66^. As zooplankton serves as a vital food source for higher trophic levels^67^, even moderate yet persistent alterations in life-history traits may have implications for freshwater ecosystem functioning under continuous exposure.

Taken together, these findings indicate that particle type and co-exposure effects vary across life stages and exposure durations. Particle-related effects dominated early responses, whereas effects associated with 2-MNQ-containing treatments became more apparent over time, particularly affecting reproductive output and body length. These results highlight that responses to single and combined stressors are not static but depend on exposure duration and organismal life stage. However, the underlying mechanisms remain unresolved. In particular, the biological availability of 2-MNQ and its interaction with particles could not be directly quantified, limiting conclusions regarding adsorption, desorption, and particle-mediated uptake pathways. Future studies should therefore integrate direct chemical analyses of particle-associated contaminants, and concentration–response approaches to better disentangle the interactions between particles and co-occurring 2-MNQ. An additional limitation of the present study might be that the reproductive output in the control group remained below the OECD 211 recommendation of 60 offspring per surviving female. However, control mortality met the validity criterion, and all treatments were conducted under identical experimental conditions. Previous chronic life-history studies in our laboratory using the same *D. magna* clone (BL2.2) have shown that reproductive performance varies with experimental conditions, particularly food availability and study design. Therefore, the present study is best interpreted in terms of relative differences among treatments rather than as a strict guideline-based toxicity classification. This interpretation is further supported by consistent treatment-related responses across multiple life-history endpoints, including body length, brood-specific reproduction, cumulative offspring production, and population growth rate (λ). Despite these limitations, the observed sublethal effects on body size and reproduction may contribute to population-level responses under prolonged exposure scenarios. Given the keystone role of daphnids in freshwater food webs, such changes may propagate through trophic interactions and contribute to alterations of ecosystem structure and functioning.

## Supplementary Information

Below is the link to the electronic supplementary material.

Supplementary Material 1

## Data Availability

All data generated or analyzed during this study are included in this published article and its supplementary information files.
