# Supplementary material for: Life-stage dependent responses to microplastics and 2-methoxy-1,4-naphthoquinone in Daphnia magna
Source: Sci Rep. 2026 Jul 20;16:22665. doi: 10.1038/s41598-026-61016-5 (PMC13385858; doi:10.1038/s41598-026-61016-5)
Supplement: Supplementary file 1 — Supplementary Material 1 [file 41598_2026_61016_MOESM1_ESM.docx]

Supplementary Material

**Life-stage dependent responses to microplastics and 2-methoxy-1,4-naphthoquinone in *Daphnia magna***

Julian Brehm^1+^, Jens G. P. Diller^1+^, Michael Schwarzer^1^, Josef Breu^2^ and Christian Laforsch^1*^

^1^Animal Ecology I, University of Bayreuth, 95447 Bayreuth & Bayreuth Center for Ecology and Environmental Research (BayCEER), 95447 Bayreuth

^2^Anorganic Chemistry I, University of Bayreuth, 95447 Bayreuth

^+^authors contributed equally

*Corresponding author: Christian Laforsch, Email: christian.laforsch@uni-bayreuth.de

*Particle size distributions*


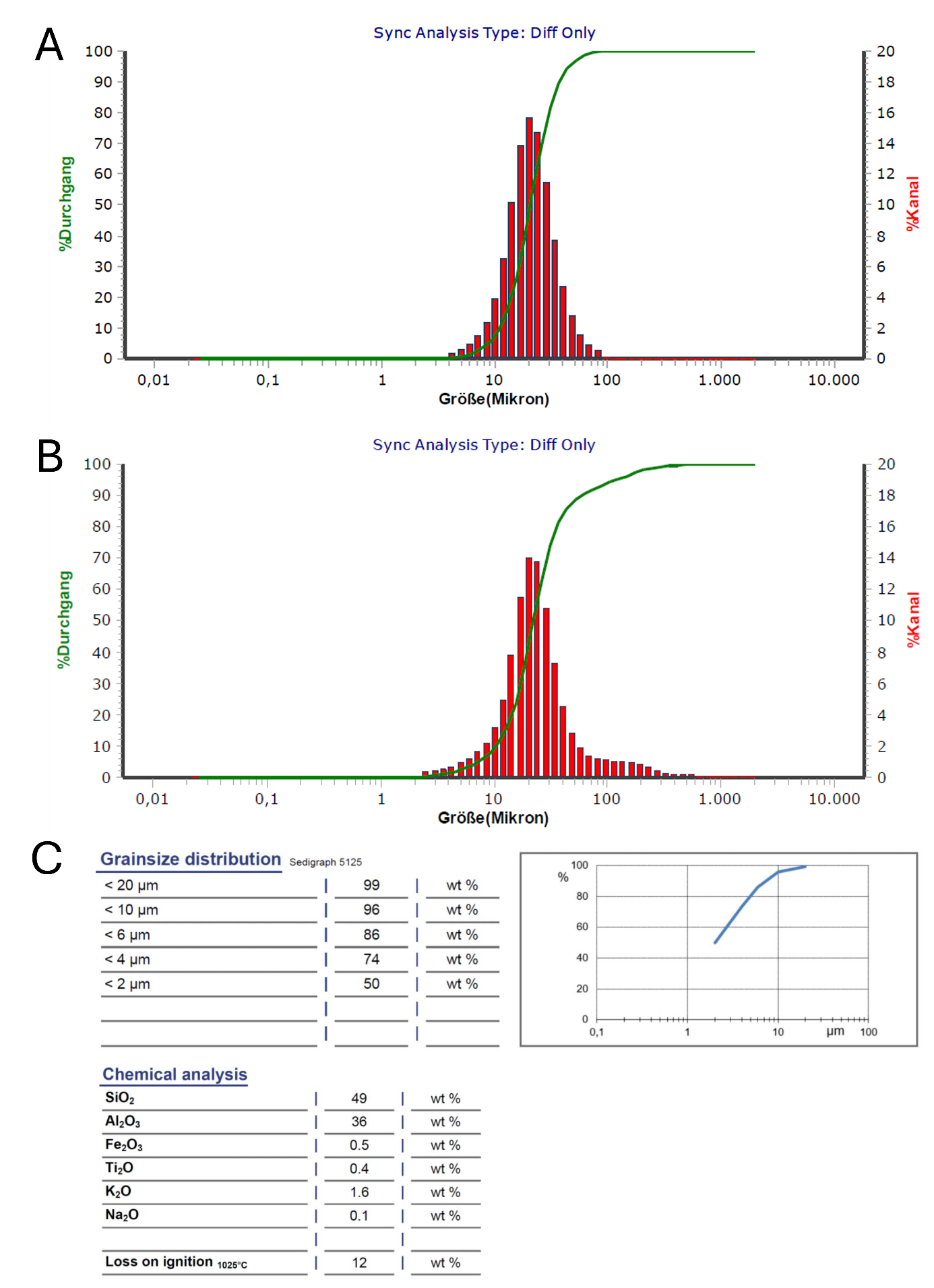


**Figure SI 1** Size distribution of used MP and control particles. (A) PS MP, d_90_: 37.54 µm, (B) PA 66 MP, d_90_: 59.15 µm, and (C) kaolinite, d_96_: <10 µm. Measurements in (A) and (B) were conducted using LD/DIA, (C) was provided by the manufacturer (Gebrüder Dorfner GmbH & CO., Hirschau, Germany).

*UV vis calibration curve and spectra of 2-MNQ*


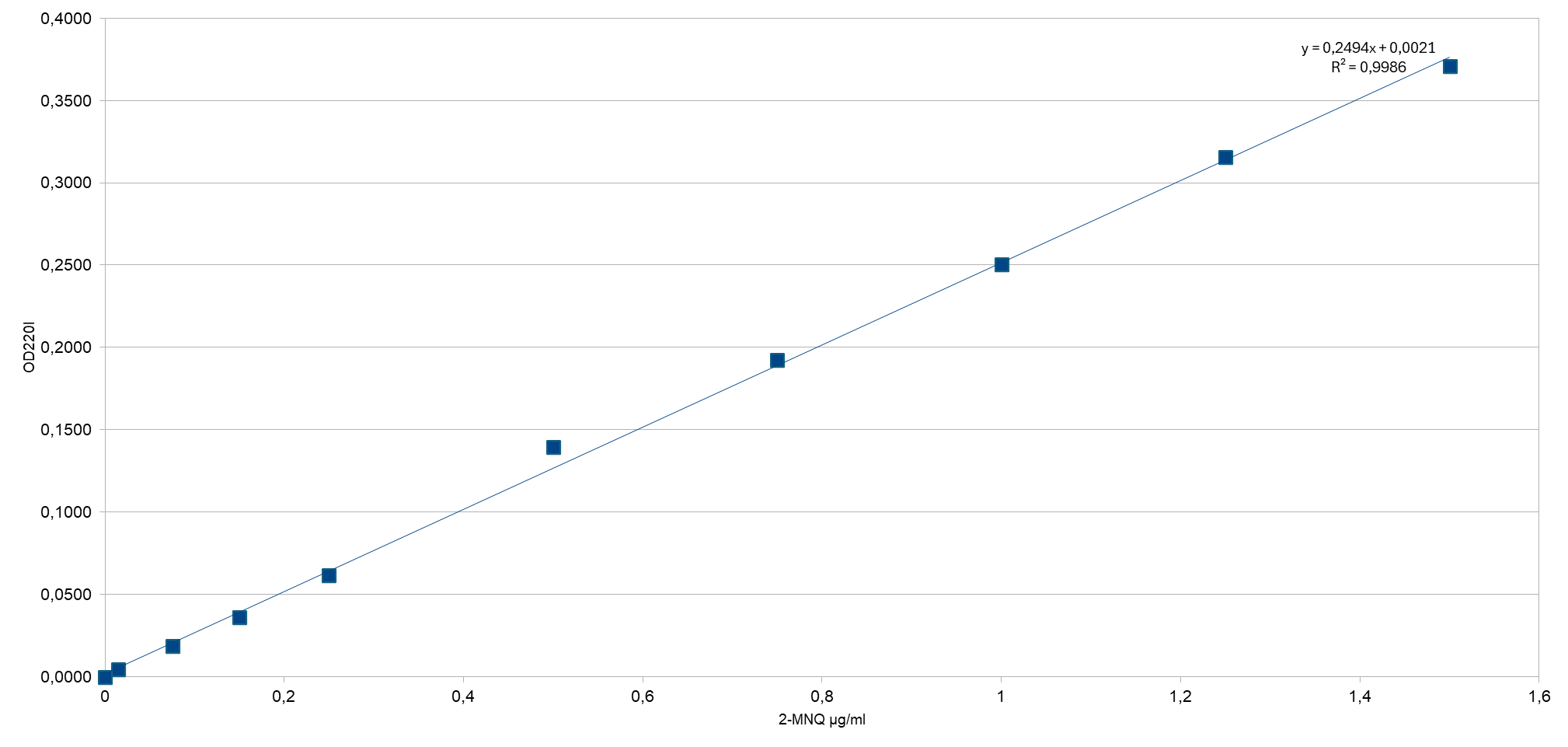


**Figure SI 2** UV-vis calibration curve at 220 nm, ranging from 0.015 to 1.5 mg 2-MNQ/L. Formula: y = 0.2494x + 0.0021; R^2^: 0.9986.


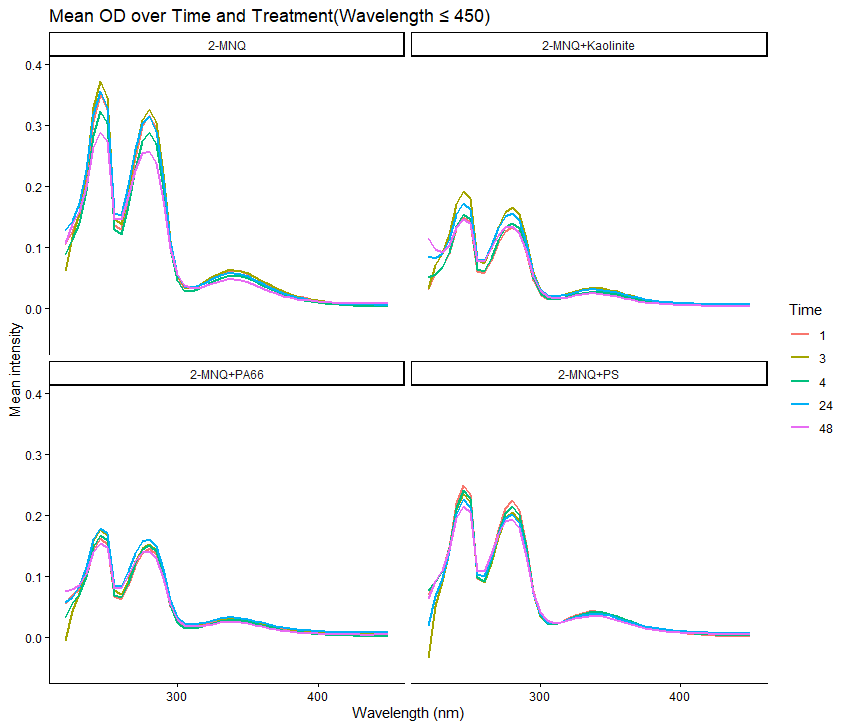


**Figure SI 3** Absorbance spectra (measured in 5 nm intervals) of 2-MNQ (10 mg/L) and 1000 P/mL in M4 of the respective treatment, blanked against M4 with 1ml/L DMSO after 1, 3, 4, 24, and 48 hours.


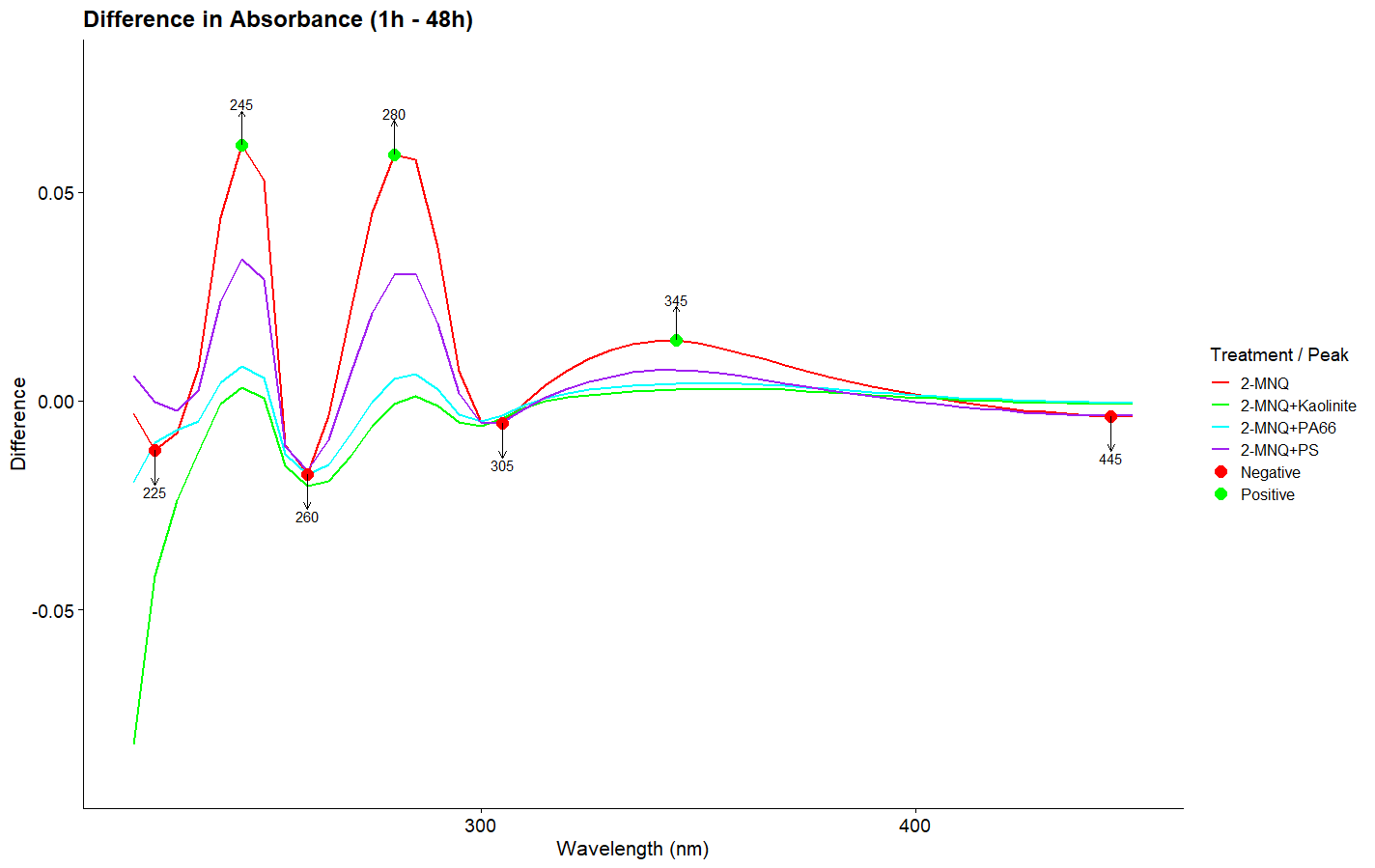


**Figure SI 4** Differences in the absorbance spectra (measured in 5 nm intervals) after 1 and 48 hours of 2-MNQ (10 mg/L) and 1000 P/mL in M4 of the respective treatment, blanked against M4 with 1ml/L DMSO. Positive peaks (green dots) at 245, 280, and 345 nm; negative peaks (red dots) at 225, 260, 305, and 445 nm.

*Life history and morphology parameters*

**Table S1** Life-history parameters of *D. magna* after chronic exposure to microplastics (MP) and 2-methoxy-1,4-naphthoquinone (2-MNQ), given as mean ± standard error (SE).
